# Herb-induced hepatitis secondary to artemisinin: A case report

**DOI:** 10.3389/fmed.2022.1033906

**Published:** 2022-10-06

**Authors:** Jiwei Zhan, Meilin Ding, Jin Li, Lei Su

**Affiliations:** ^1^Department of Geriatrics, Central People's Hospital of Zhanjiang, Zhanjiang, China; ^2^Department of Geriatrics, First Affiliated Hospital of Sun Yat-sen University, Guangzhou, China

**Keywords:** artemisinin, drug-induced liver injury, cholestatic hepatitis, jaundice, plasma exchange (PE)

## Abstract

This report describes a rare case of severe liver injury secondary to a herbal supplement containing artemisinin. The patient received plasma exchange with bilirubin filtration adsorption therapy. The case is unique in its severe cholestasis. Bilirubin decreased to baseline after 62 days. This will help to improve clinicians' awareness of the diagnosis and treatment of drug-induced liver injury.

## Case introduction

A 55-year-old-male was admitted due to dark urine and jaundice. Before admission, he took two capsules of artemisinin extract (containing 200 mg artemisinin extract + 10 mg alkaloid) per day for 1 month for shoulder pain. Gradually, he developed symptoms such as loss of appetite, tiredness of oil and itchy skin. He had no abdominal pain, vomiting, fever, diarrhea, etc. He has no history of hypertension, diabetes, hepatitis and tuberculosis. He did not smoke or drink alcohol. Physical examination revealed tachycardia. His BMI was normal (22.22 kg/m^2^).

Abnormal liver function was found on admission (e.g., serum alanine aminotransferase of 908 IU/L [normal: 10–55 IU/L], total bilirubin 151.0 umol/L [normal: 3–22 IU/L], direct bilirubin 83.4 umol/L [normal: 0.5–7 IU/L], indirect bilirubin 83.4 umol/L [normal: 3–15 IU/L], blood ammonia 40 umol/L [normal: 9-−33 IU/L]. International normalized ratio (INR) was normal. His glycated hemoglobin (HBA1c) was high (8.70%). Tests for hepatitis A, B, C, D, E, and Epstein-Barr virus were negative. Alpha-fetoprotein, carbohydrate antigen 19–9, ceruloplasmin, and antinuclear antibody ANA were negative. Anti-mitochondrial M2 antibody was weakly positive (±). Laboratory tests did not support known infection (white blood cell, procalcitonin, cytomegalovirus CMV-DNA, human immunodeficiency virus type I type II, Treponema pallidum antibody were all negative).

**Figure 1:**
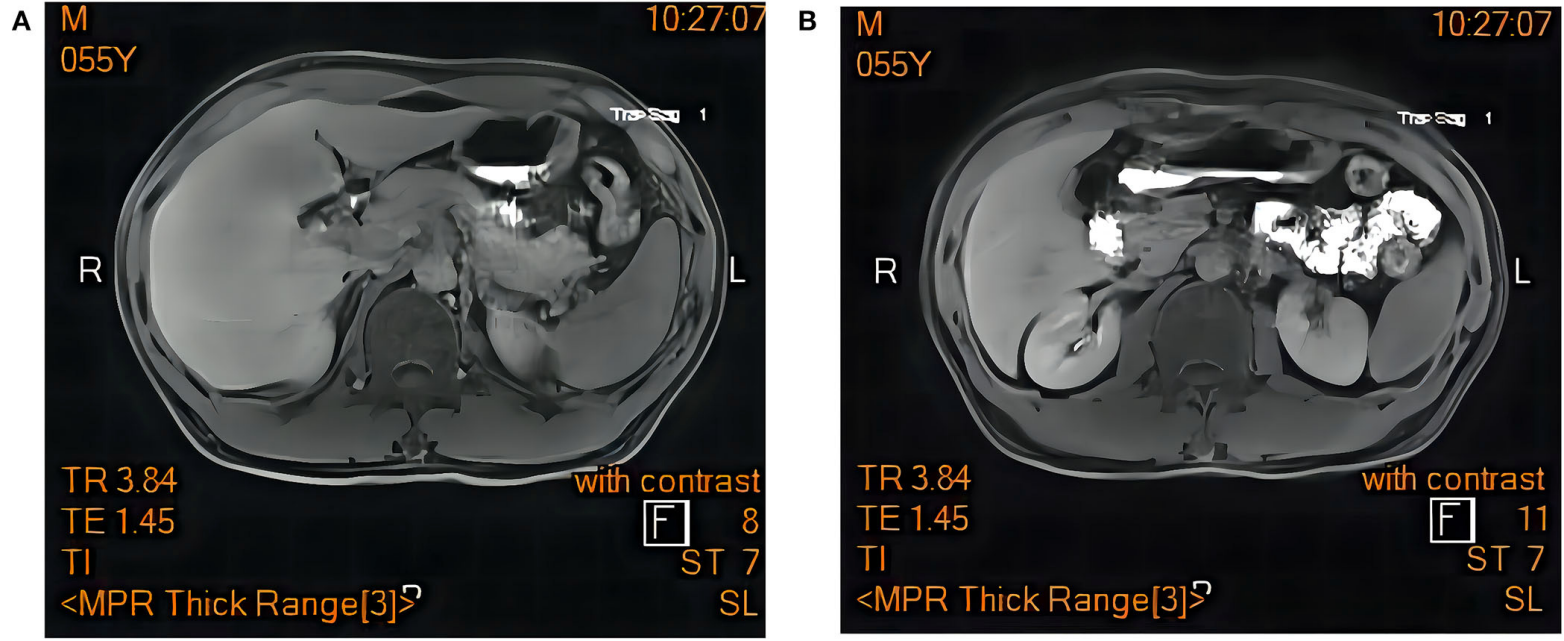
Magnetic resonance cholangiopancreatography of upper abdomen showed a decreased liver uptake and bile duct excretion of Gd-EOB-DTPA. **(A)** hepatobiliary phase. **(B)** excretion period.

**Figure 2:**
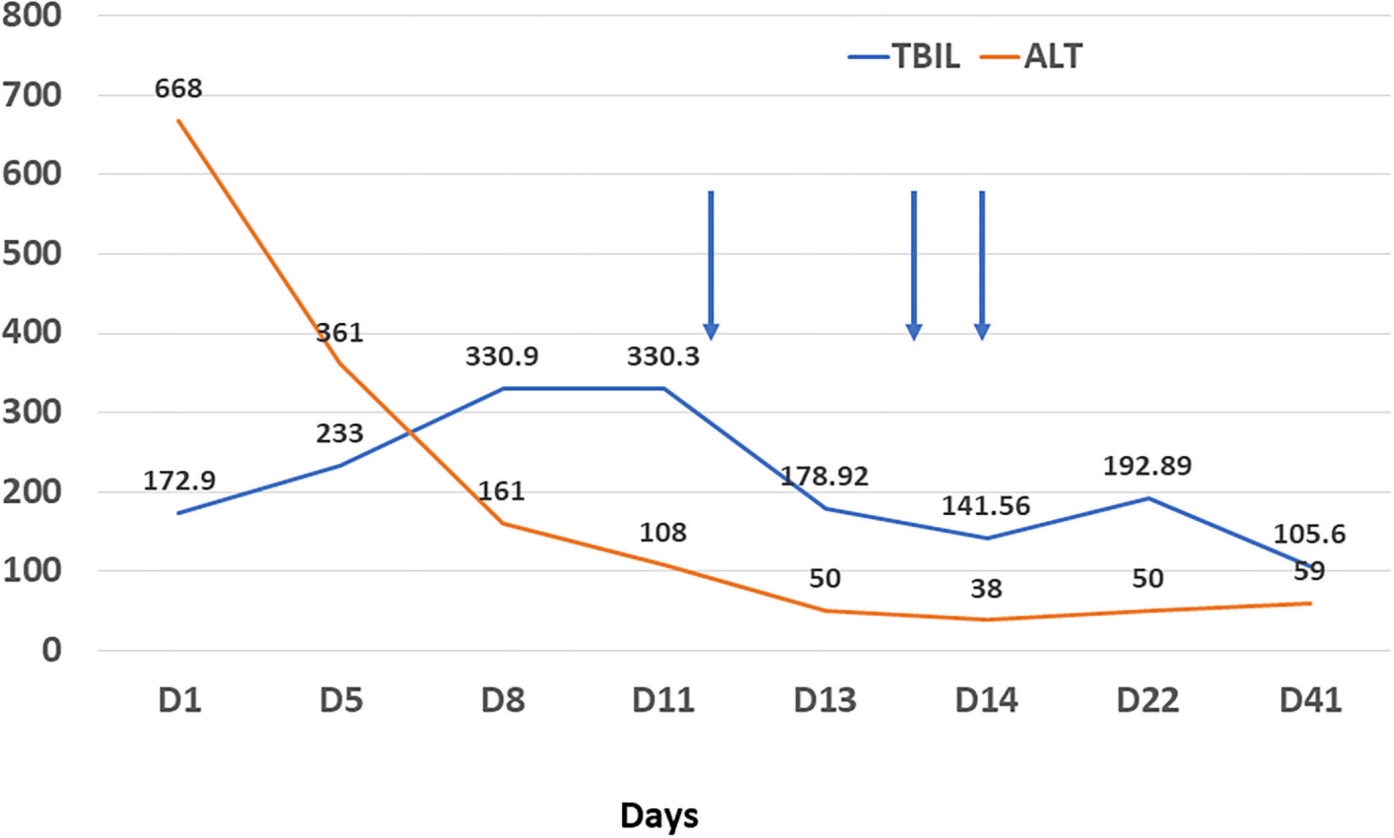
Changes of alanine aminotransferase (orange line, U/L) and total bilirubin (blue line, umol/L) during treatment. Arrow refers to plasma filtration adsorption.

Resonance Cholangiopancreatography of upper abdomen showed a decreased liver uptake and bile duct excretion of Gd-EOB-DTPA, suggesting liver function impairment (Figure 1). He was diagnosed with drug-induced liver injury, hepatocellular injury type (R ratio 9.3), acute, RUCAM eight points (very likely), severity level three. After admission, polyene phosphatidylcholine, reduced glutathione, ursodeoxycholic acid capsules, and insulin were given. Bilirubin continued to rise after artemisinin was discontinued. On day 12, 14, and 18 after admission, plasma exchange plus bilirubin adsorption therapy was performed three times. His symptoms improved and discharged on day 22. Bilirubin decreased to baseline after 40 days (Figure 2).

## Discussion

This patient was diagnosed with drug-induced liver damage, based on the following criteria (1, 2): (1) There was a clear history of taking artemisinin before the liver function impairment is found. ALT ≥ 5 × upper limit normal (ULN). ALP ≥ 2 × ULN, accompanied by increased gamma-glutamyl transferase (GGT). Total bilirubin ≥ 2 × ULN. (2) Laboratory tests can rule out other causes of liver damage, such as viruses, immunity, alcohol, bile ducts, blood vessels, etc. (3) Roussel Uclaf Causality Assessment Method score was 8 points. (4) Other medications with clear toxicity were excluded. (5) We were able to obtain and verify the remaining medicinal materials, prescription composition, usage and dosage. Regrettably, the patient refused to undergo liver biopsy to further confirm the diagnosis and assess the severity of liver damage.

Artemisinin and its derivatives have been widely used to treat malaria. It also demonstrate antiparasitic, antibacterial, anticancer, and anti-fibrotic effects (3). However, report of severe liver injury is rare. Malhotra reported a 52-year-old man with drug-induced liver injury after taking artemisinin extract for 1 week (4). He had elevated alanine aminotransferase. Liver function returned to normal after 14 days. Our case is unique in prolonged cholestatic phase. Clinical manifestation of our case is consistent with previous report by Shiva Kumar (5). It took 60 days for bilirubin to return to normal. We ended up having to resort to plasma exchange. The mechanism is unclear.

Lobular inflammation (5) and lymphocytic infiltration of the bile ducts (6) have been found on live biopsy.

The reason may be related to the patient's off-label dosage or low cytochrome P450 (CYP2B6) activity (7).

In most cases, liver injury can be improved after drug withdrawal. However, in our case bilirubin only decreased after plasma exchange. The liver assist systems can provide an effective “bridge” to recovery of liver function. However, more clinical trials are needed to validate the efficacy (8).

Attention should be paid to the hepatotoxicity of herbal supplements. It is important to avoid improper use of herbal products in the community. Further studies are needed to identify host-related risk factors for herb-Induced Liver Injury.

## Data availability statement

The original contributions presented in the study are included in the article/supplementary material, further inquiries can be directed to the corresponding author.

## Ethics statement

The studies involving human participants were reviewed and approved by Ethics Committee of First Hospital of Sun Yat-Sen University. The patients/participants provided their written informed consent to participate in this study.

## Author contributions

LS designed and drafted the work, and substantively revised it. JZ has made contributions to acquisition of data and drafted the work. JL and MD has made contributions to acquisition of data and drafted the work. All authors contributed to the article and approved the submitted version.

## Funding

LS was supported by National Nature Science Foundation of China (81701367).

## Conflict of interest

The authors declare that the research was conducted in the absence of any commercial or financial relationships that could be construed as a potential conflict of interest.

## Publisher's note

All claims expressed in this article are solely those of the authors and do not necessarily represent those of their affiliated organizations, or those of the publisher, the editors and the reviewers. Any product that may be evaluated in this article, or claim that may be made by its manufacturer, is not guaranteed or endorsed by the publisher.

## Abbreviations

ALT: alanine aminotransferase
RUCAM: Roussel Uclaf Causality Assessment Method
ULN: upper limit normal
GGT: gamma-glutamyl transferase.
